# Electron shuttle-dependent biofilm formation and biocurrent generation: Concentration effects and mechanistic insights

**DOI:** 10.3389/fmicb.2023.1070800

**Published:** 2023-03-01

**Authors:** Xiao Zhu, Fei Dou, Mingliang Long, Xinxin Wang, Wei Liu, Fangbai Li, Tongxu Liu, Yundang Wu

**Affiliations:** ^1^Guangzhou Institute of Geochemistry, Chinese Academy of Sciences, Guangzhou, China; ^2^National-Regional Joint Engineering Research Center for Soil Pollution Control and Remediation in South China, Guangzhou, China; ^3^Guangdong Key Laboratory of Integrated Agro-environmental Pollution Control and Management, Institute of Eco-environmental and Soil Sciences, Guangdong Academy of Sciences, Guangzhou, China; ^4^University of Chinese Academy of Sciences, Beijing, China; ^5^College of Materials and Energy, South China Agricultural University, Guangzhou, China

**Keywords:** electron shuttle, extracellular electron transfer (EET), biofilm, *Shewanella oneidensis* MR-1, electroactive bacteria (EAB)

## Abstract

**Introduction:**

Electron shuttles (ESs) play a key role in extracellular electron transfer (EET) in *Shewanella oneidensis* MR-1. However, the quantification relationship between ES concentration, biofilm formation, and biocurrent generation has not been clarified.

**Methods:**

In this study, 9,10-anthraquinone-2-sulfonic acid (AQS)-mediated EET and biofilm formation were evaluated at different AQS concentrations in bioelectrochemical systems (BESs) with *S. oneidensis* MR-1.

**Results and discussion:**

Both the biofilm biomass (9- to 17-fold) and biocurrent (21- to 80-fold) were substantially enhanced by exogenous AQS, suggesting the dual ability of AQS to promote both biofilm formation and electron shuttling. Nevertheless, biofilms barely grew without the addition of exogenous AQS, revealing that biofilm formation by *S. oneidensis* MR-1 is highly dependent on electron shuttling. The biofilm growth was delayed in a BES of 2,000 μM AQS, which is probably because the redundant AQS in the bulk solution acted as a soluble electron acceptor and delayed biofilm formation. In addition, the maximum biocurrent density in BESs with different concentrations of AQS was fitted to the Michaelis–Menten equation (*R*^2^ = 0.97), demonstrating that microbial-catalyzed ES bio-reduction is the key limiting factor of the maximum biocurrent density in BESs. This study provided a fundamental understanding of ES-mediated EET, which could be beneficial for the enrichment of electroactive biofilms, the rapid start-up of microbial fuel cells (MFCs), and the design of BESs for wastewater treatment.

## Introduction

Extracellular electron transfer (EET) is a key process involved in microbial anaerobic respiration (Shi et al., 2016; Wu et al., 2016; Kumar et al., 2017). Microbes with EET ability can transfer electrons produced by intracellular metabolism to extracellular solid-state electron acceptors. These can be used to construct microbial fuel cells (MFCs) (Wang et al., 2009; Yuan et al., 2013; Li et al., 2018; Logan et al., 2019) and to design bioelectrochemical systems (BESs) for wastewater treatment (Feng et al., 2008; Liu et al., 2014; Wan et al., 2020). Therefore, EET has received widespread attention in the past decades.

Electron shuttles (ESs) are chemicals that can enhance the rate of EET through redox cycling (Hernandez and Newman, 2001; Watanabe et al., 2009; Brutinel and Gralnick, 2012; Glasser et al., 2017). Although the importance of ES-mediated electron shuttling in natural systems has been recognized, the role of ES in artificial BESs has remained controversial for a long time. Researchers generally agree that wildly existing humic substances in natural environments can markedly influence environmental processes, such as iron reduction and methane emissions in soil and sediments, *via* electron shuttling (Lovley et al., 1996; Klüpfel et al., 2014; Qiao et al., 2019). Nevertheless, some researchers believe that the multi-step reaction involving the mediation of ES causes high potential losses, which limits the use of ES in artificial MFCs (Torres et al., 2010). However, our recent study demonstrated that exogenous ESs substantially enhanced biofilm formation in *Shewanella oneidensis* MR-1 (15- to 36-fold) by forming a microenvironment with sufficient oxidized ESs as soluble electron acceptors (Wu et al., 2020). This discovery revealed the importance of ES as a biofilm regulator in artificial BESs; in other words, ES can be potentially used as a regulator to carry out a rapid start-up of MFCs; however, the regulating mechanism remains unclear.

Electron shuttle concentration should be a critical parameter in biofilm formation. Although empirical models have been used to describe ES-mediated biocurrent generation at low ES concentrations (Picioreanu et al., 2007), to the best of our knowledge, there have been no quantification studies on the relationship between ES concentration, biofilm enhancement, and increased biocurrent generation over a wide concentration range. Low concentrations of ES facilitate electron shuttling (Wu et al., 2018, 2019); however, at high concentrations, ES may act as an electron acceptor before it is fully reduced. *S. oneidensis* MR-1 may prefer soluble electron acceptors to solid electron acceptors; thus, a high concentration of soluble electron acceptors may competitively suppress electron transfer from microbes to the electrode. In addition, as diffusion is a key process in electron shuttling (Torres et al., 2010), increasing the EM concentration may enhance the diffusion process and increase the EET rate, which may also influence biofilm growth and biocurrent. However, with an increase in ES concentration, the balance between the beneficial effects of increased diffusion and the detrimental effects of competitive suppression is unclear. The physicochemical constraints of shuttle-mediated biocurrent generation at different ES concentrations need to be clarified.

Therefore, it is necessary to quantify the biocurrent and biofilms under different ES concentrations and to use electron transfer models to quantitatively analyze the relationship between ES concentration, biocurrent generation, and biofilm enhancement. A model ES, 9,10-anthraquinone-2-sulfonic acid (AQS) (O'Loughlin, 2008; Wolf et al., 2009; Wu et al., 2014), was used in this study. Bioelectrochemical systems with *S. oneidensis* MR-1 and different concentrations of AQS were constructed (Wu et al., 2018; Qin et al., 2020). This study aimed to examine the effects of AQS concentration on biocurrent generation and biofilm formation, determine the mechanisms by which AQS concentration affects these factors, and identify the physicochemical limitations on the maximum biocurrent density and biofilm biomass in an AQS-mediated EET system. The findings of this study will provide a theoretical basis for understanding the regulation of biofilm formation in artificial BESs and the ecological effects of ES in natural environments.

## Materials and methods

### Materials and cell growth

*Shewanella oneidensis* MR-1, a well-known metal-reducing bacteria, was purchased from the Marine Culture Collection of China (China) (Hau and Gralnick, 2007). The strain was aerobically cultured in Luria–Bertani medium at 30°C in a shaker (180 rpm). Next, it was centrifuged, washed, and diluted to the desired concentration for subsequent bioelectrochemical experiments. 9,10-Anthraquinone-2-sulfonic acid (AR, 98.0%) was obtained from Acros (Belgium), and all other chemicals were purchased from Guangzhou Chemical Reagent Factory (Guangzhou, China).

### BES setup and electrochemical measurements

The BES was constructed in a glass media bottle, which was sealed with silicone mats and a hot-melt adhesive to keep the system anaerobic (Supplementary Figure 1). Three electrodes were equipped on the cap, with carbon clothes (2 × 2 cm) as working and counter electrodes and calomel electrodes as reference electrodes. Furthermore, 110 ml of MR-1 suspension (OD_600_ = 1.0) was cultivated in the BES in the presence of 30 mM lactate with different concentrations of AQS. The medium contained 200 mM of phosphate-buffered saline (pH = 7), NH_4_Cl (1.24 g·L^−1^), KCl (0.52 g·L^−1^), vitamin stock solution (5 ml·L^−1^), and mineral stock solution (12.5 ml·L^−1^) (Supplementary Table 1). A fixed potential of 0.441 V (vs. SHE) was applied to the BES, which was controlled by a potentiostat (CHI1040C; Chenhua Co., Ltd., Shanghai, China). A slow-scan cyclic voltammetry (CV) (1 mV·s^−1^) was performed after the potentiostatic incubation.

### Microbiological measurements

A scanning electron microscope (SEM, ProX, Phenom, Netherlands) was used for observation of biofilms on the electrode. The biofilms in the BES were incubated under potentiostatic conditions for 4 days, and after a subsequent cyclic voltammetry (CV) scan, the biofilm samples were obtained by cutting off the electrodes. Electrode samples were first washed in a 0.1 M phosphate buffer solution (pH 7.0) for 5 min, followed by hardening in 2.5 % glutaraldehyde solution for 5 h. Next, the samples were dehydrated in an ethanol gradient (10%, 30%, 50%, 70%, 90%, 95%, and 100%) and t-BuOH. Finally, after freeze-drying, the samples were coated with evaporated platinum before being viewed in the SEM with an operating voltage of 15 kV. The protein in the cells on the electrodes was dissolved and extracted using 0.2 M NaOH (Qin et al., 2020), followed by quantification with Coomassie blue staining using a protein quantification kit (C503041-1000 Modified Bradford Protein Assay Kit; Sangon Biotech, Shanghai, China).

### Special treatments of the BES

Two special processing conditions were used in this study for the biocurrent density test. The first was the pre-reduction of AQS in the BES. The reducing kinetic of AQS at its different concentrations was first examined *in situ* using a UV–Visible diffuse-transmittance spectrometer (TU-1901, equipped with an integrating sphere; Persee Co., Ltd., Beijing, China) (Wu et al., 2014, 2019). The absorption of AH_2_QS at 430 nm was used for quantification because for AQS and sodium hyposulfite, no absorption was observed at 430 nm, and the absorption of the bacteria was stable at 430 nm (Supplementary Figure 2). As shown in Supplementary Figure 3, 1,000 μM of AQS can be reduced by the cells within 1 day. Hence, new BESs with different AQS concentrations were constructed but kept open circuit for 4 days, thus ensuring that the AQS in the BESs was all reduced. Next, fresh cells (OD_600_ = 1.0) and 10 mM of lactate were added to the system, and 0.441 V vs. SHE was applied to the electrode to assess biocurrent generation. Under the second special treatment, the biofilms were all incubated in the BES with 50 μM AQS for 4 days. The original medium in the BES was then replaced with a new medium containing different concentrations of pre-reduced AQS.

## Results

### Effects of AQS concentration on biocurrent generation

**Figure 1:**
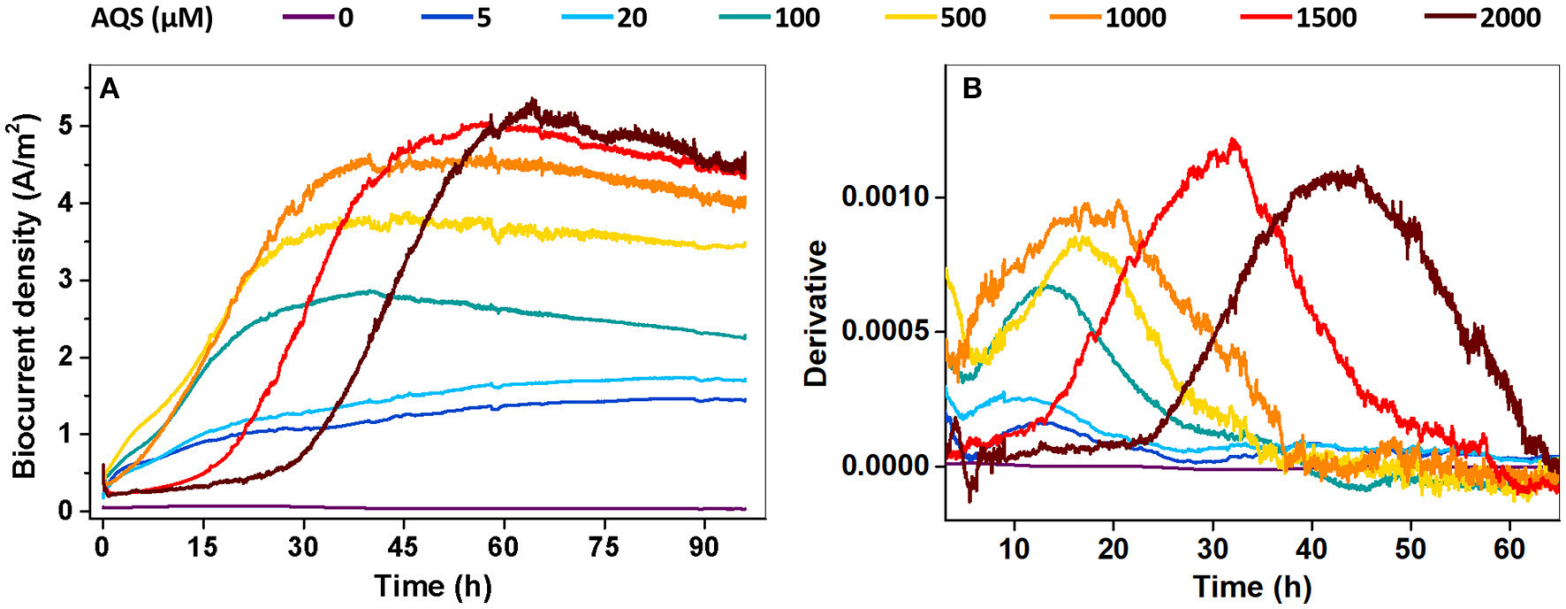
**(A)** Biocurrent density vs. time at different concentrations of 9,10-anthraquinone-2-sulfonic acid (AQS) (0–2,000 μM); **(B)** first derivative analysis of the biocurrent results in panel **(A)**.

Biocurrent generation in the BES was monitored at different AQS concentrations. The results show that the biocurrent of each BES gradually increased at the very beginning and then rapidly increased to a constant output in approximately 48 h (Figure 1A). Biocurrent outputs were markedly enhanced by AQS compared with those in the treatment without EM. The maximum biocurrent (*I*_max_) increased with a rise in AQS concentration from 0 to 1,500 μM, and remained constant with a continued increase in AQS concentration from 1,500 to 2,000 μM. A first derivative analysis of biocurrent vs. time, based on the data in Figure 1A, is shown in Figure 1B. The peak position, which represented the mid-log phase of the increase in biocurrent, shifted positively as the AQS concentration increased from 100 to 2,000 μM.

### Effects of AQS concentration on biofilm and planktonic cell growth

**Figure 2:**
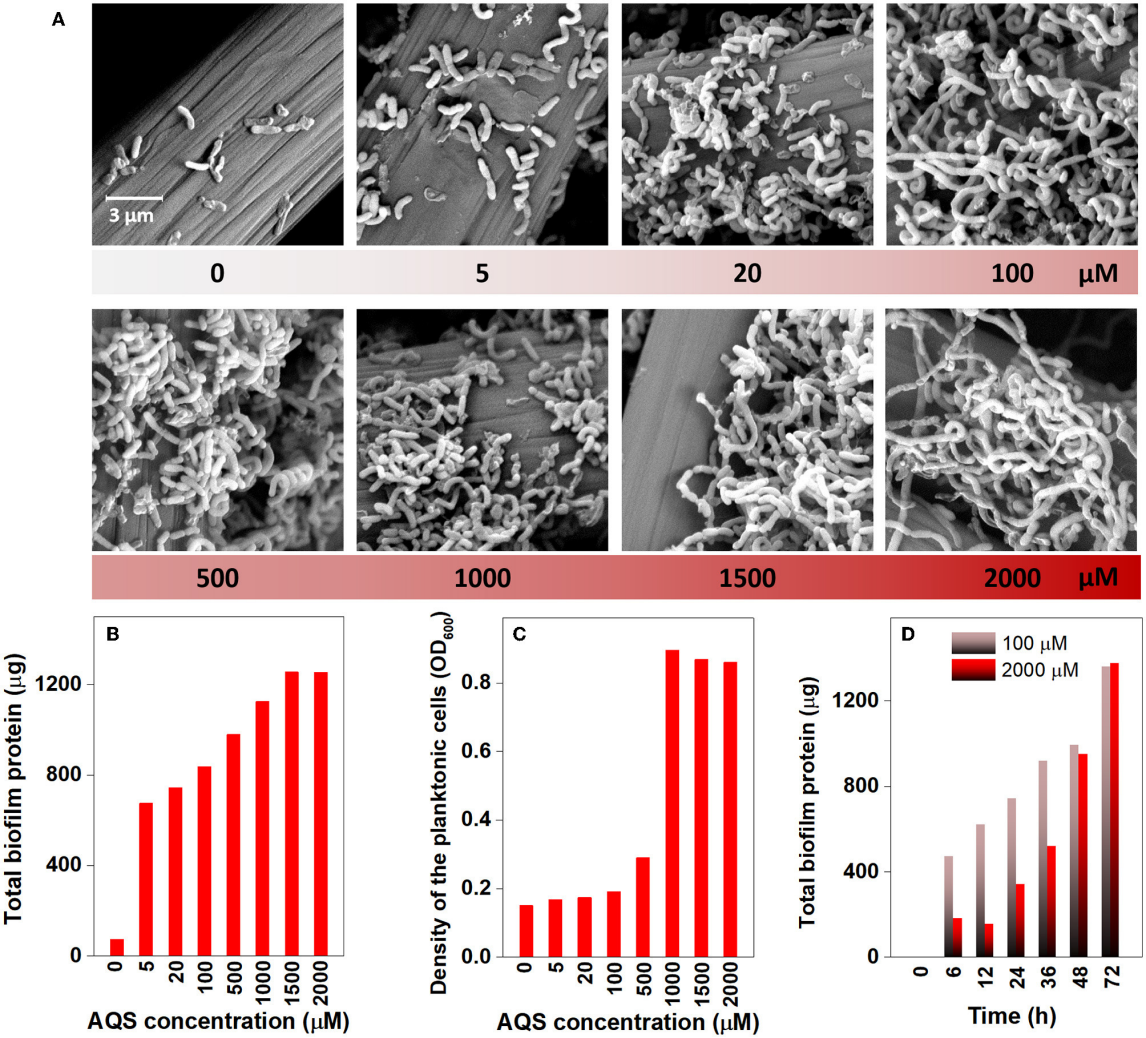
Scanning electron microscopy images of the biofilm on electrodes **(A)**, total biofilm protein on electrodes **(B)**, and cell density in suspension **(C)** at different concentrations of 9,10-anthraquinone-2-sulfonic acid (AQS) at the end of day 4. **(D)** Biofilm quantification with time in BESs with 100 and 2,000 μM AQS.

The morphology of the biofilm on the electrode was examined *via* SEM on day 4 (Figure 2A). It is relatively clear that very few cells grew on the electrode in the BES without AQS, whereas the number of cells on the electrode dramatically increased with increasing concentrations of AQS from 5 to 100 μM. For accurate quantification, the total protein of the biofilm was extracted and quantified to indicate the changes in biofilm biomass (Figure 2B). Similar to the results of SEM analysis, the total protein of the biofilm increased from 74 to 836 μg (11-fold) with an increase in AQS from 0 to 100 μM and then slightly increased from 836 to 1,253 μg with a continued increase of AQS from 1,000 to 2,000 μM. The cell density in the suspension was also measured at the end of day 4. Figure 2C shows that the OD_600_-values of BESs at 0–100 μM AQS were similar; however, the value markedly increased as the AQS concentration increased from 500 to 2,000 μM. In addition, the results in Figure 2D show that the biofilm total protein in the BES of 2,000 μM before 36 h was much lower than that in the BES of 100 μM, which proved that biofilm growth was delayed in the system with a high concentration of AQS.

### Biofilm electrochemical properties in BESs with different AQS concentrations

**Figure 3:**
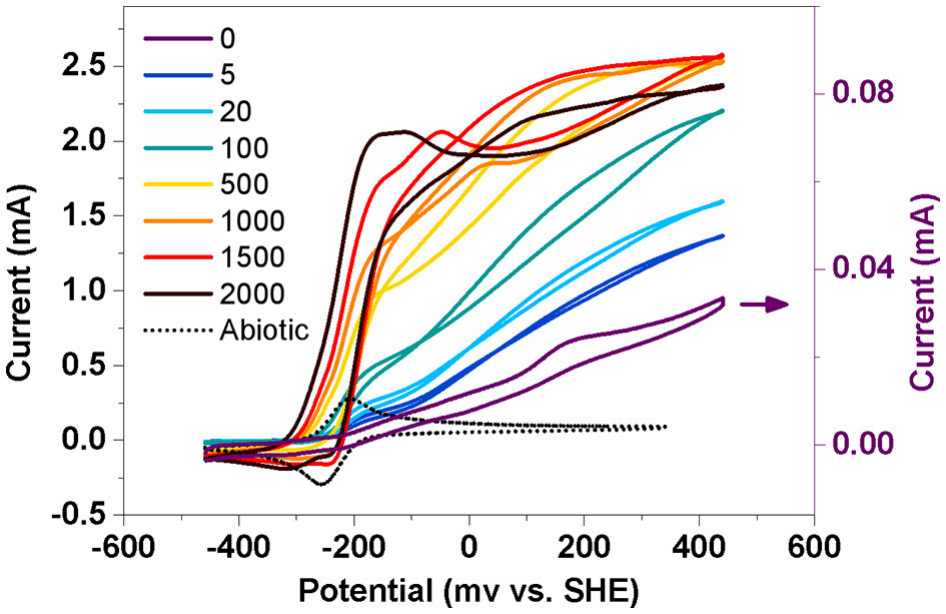
Slow-scan cyclic voltammetry of biofilm in a bioelectrochemical system (BES) at different AQS concentrations. Abiotic represents a electrochemical system with 50 μM AQS but without cells.

To further analyze the electrochemical properties of the biofilm, a slow-scan CV test was performed after 4 days of potentiostatic incubation (Figure 3). Only one pair of peaks was obtained in the BES without AQS (0 μM) over the scanned potential of −459 to 241 mV. Nevertheless, two batches of notable signals were observed in the AQS-mediated systems. The first was a sigmoid-shaped catalytic current at approximately −200 mV, which was consistent with the peak position of pure AQS; meanwhile, the peak height increased with an increase in the AQS concentration from 0 to 2,000 μM. The other was a pair of redox peaks at approximately 0–200 mV, which was consistent with the peak position of the biofilm in the BES without AQS.

### AQS-mediated biocurrent generation under special processing conditions

**Figure 4:**
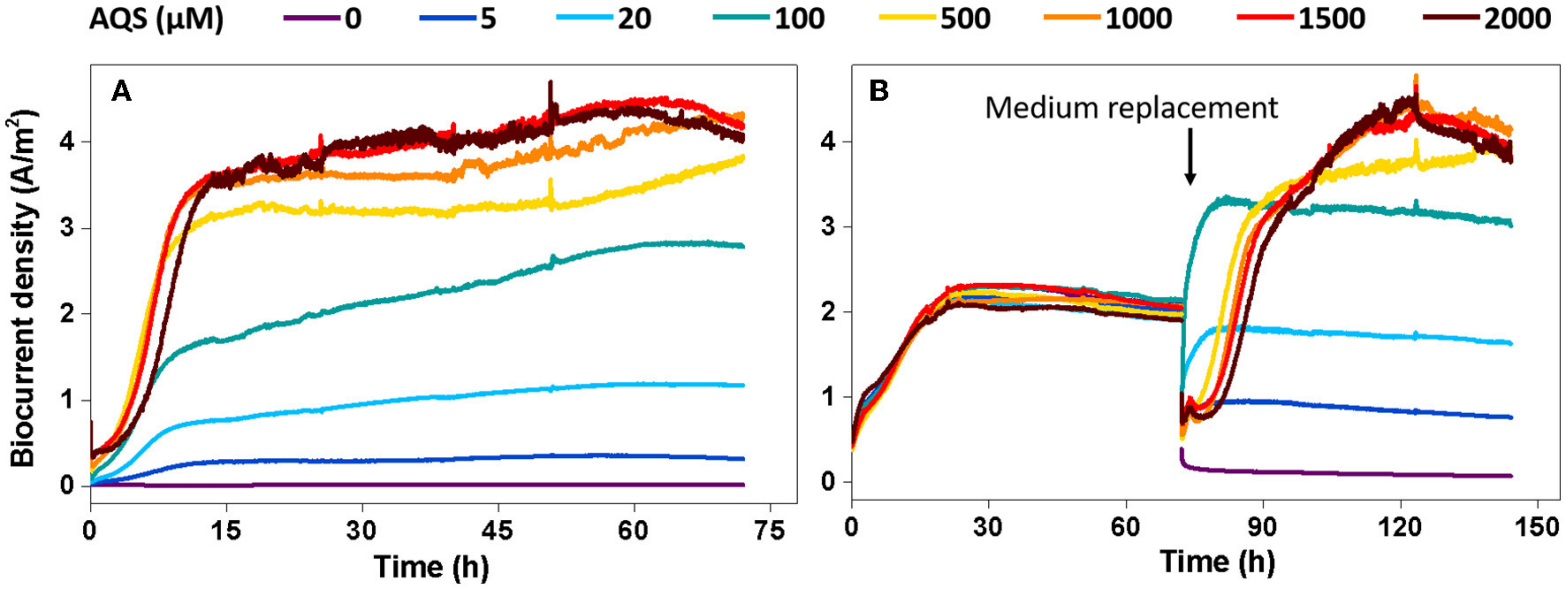
Biocurrent density vs. time under two special conditions. **(A)** Potentiostatic incubation in the bioelectrochemical system (BES) was initiated after 9,10-anthraquinone-2-sulfonic acid (AQS) pre-reduction. **(B)** The biofilms were incubated in BES with 50 μM AQS for 4 days, after which the original medium in the BES was replaced with a new medium containing different concentrations of pre-reduced AQS.

To further analyze the possible maximum biocurrent generated by AQS-mediated EET, the biocurrent was monitored under two special processing conditions. The purpose of the first condition was to eliminate the inhibitory effect of high concentrations of AQS on biofilm formation. The BESs were kept open circuit for 96 h to pre-reduce AQS, and subsequently, fresh bacteria were supplemented to the BES and cultured under potentiostatic conditions. Under this condition, AQS was reduced, and thus, it could not act as an electron acceptor to compete with the electrode for electrons. The analysis of biocurrent vs. time is shown in Figure 4A. The biofilm biomass in each BES is shown in Supplementary Figure 5. The *I*_max_ increased with an increase in the AQS concentration from 0 to 2,000 μM. The first derivative analysis of the biocurrent is shown in Supplementary Figure 4. The delay of the mid-log phase in Supplementary Figure 4 disappeared, compared with the delay shown in Figure 1B.

The second condition was to eliminate the influence of the biofilm differences on biocurrent production as much as possible. Under the second condition, all BESs were constructed using 50 μM AQS and cultured under potentiostatic conditions for 4 days to form a mature biofilm. Next, the medium in each BES was replaced with a new medium containing different concentrations of pre-reduced AQS to assess biocurrent generation. Under this condition, the difference in biofilm biomass at different AQS concentrations was low (Supplementary Figure 6). The biocurrent density is shown in Figure 4B. *I*_max_ increased continuously with an increase in AQS concentration from 0 to 2,000 μM; however, the increase slowed down, and *I*_max_ reached a maximum value when the AQS concentration was >500 μM.

## Discussion

### Dependence of biofilm formation on AQS concentration

**Figure 5:**
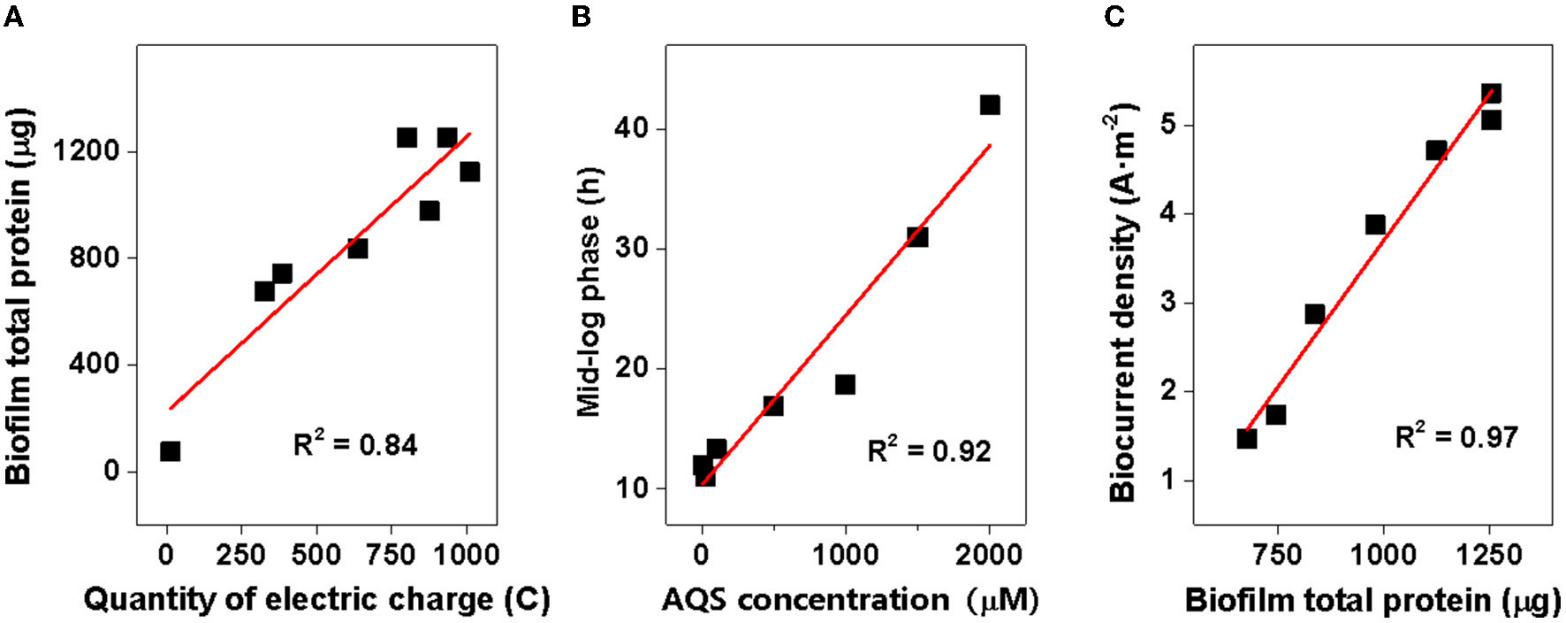
Correlation analysis. **(A)** Total protein of biofilms vs. quantity of electric charge; **(B)** time of mid-log phase vs. 9,10-anthraquinone-2-sulfonic acid (AQS) concentration; and **(C)** biocurrent density vs. biofilm total protein.

The results of this study showed a close relationship between the growth of biofilm and AQS concentration. As shown in Figure 2B, biofilm total protein content increased by 9- to 17-fold with the addition of AQS, indicating the significant role of shuttling in the formation of biofilm. This appropriately explains the phenomenon reported in previous studies. The slow growth of MR-1 biofilms in some previous studies may be attributed to a lack of exogenous ESs (Bretschger et al., 2007). Conversely, some later studies showed adequate growth of MR-1 biofilms without the use of exogenous shuttles. This was probably because shuttle-like components, such as yeast extract, which contains flavin, were added to the culture medium (Okamoto et al., 2014) or the cells were pre-cultured in fumarate, which can endogenously produce flavin (Marsili et al., 2008). In the present study, MR-1 could hardly proliferate on the electrode surface in the system in the absence of AQS, suggesting a strong dependence of MR-1 biofilm formation on soluble ES. In addition, a positive correlation was observed (*R*^2^ = 0.84) between the total protein and the quantity of electric charge in the biofilms (Figure 5A), suggesting that the increase in biomass contributed substantially to the enhancement of biocurrent generation. Although ES is critical to MR-1, the results of this study showed that the excessive addition of AQS did not continuously increase the biofilm biomass. The *R*^2^-value in Figure 5A is only 0.84. The standardized residual reached 117. This is probably because, when the biofilm reaches a certain thickness, it becomes saturated and it is difficult to continue to proliferate. Therefore, after the biofilm mass reaches a certain level, the linear relationship between biofilm mass and the quantity of electric charge is weakened.

In addition, the mid-log phase represented by the first derivative data was delayed at these high concentrations (Figure 1B), implying a delayed biofilm formation as AQS concentration increased. This conclusion has been further confirmed by the strong linear correlation between the mid-log phase and AQS concentration (*R*^2^ = 0.85, standardized residual = 1.68) (Figure 5B) and the biofilm total protein measured with time in BESs of 100 and 2,000 μM (Figure 2D). Coupling the delay of biofilm formation with the higher OD-value of the suspension at high AQS concentrations (Figure 2C), a hypothesis can be generated, wherein the added oxidized AQS at a high concentration, as a soluble electron acceptor, is the key factor accounting for delayed biofilm formation. As shown in previous studies, *S. oneidensis* MR-1 will lysis under electron acceptor-limited conditions (Liu et al., 2020), and released nutrients and extracellular DNA (eDNA) from cell lysis have confirmed its contribution to biofilm formation (Gödeke et al., 2011; Binnenkade et al., 2014). In this study, AQS oxidized by the electrode could only be used by the cells near the electrode due to diffusion limitation. Once oxidized AQS in bulk solution is fully reduced, and planktonic cells away from the electrode will face the stress of lacking the electron acceptor, in turn leading to lysis. High-concentration AQS needs more time to be fully reduced, which could delay the lysis of the planktonic cells. As shown in Figure 2C, the OD of the suspension decreased from 1.0 to 0.19 in the system with 100 μM AQS (low concentration) after 4 days of incubation, but the OD in the system with 2,000 μM AQS (high concentration) remained at 0.86. A higher proportion of cell lysis at low concentrations results in a larger amount of eDNA and nutrient release, probably favoring the early formation of biofilm. In other words, high-concentration AQS decreased the cell lysis, thus probably delaying biofilm formation. To further evaluate the effect of added oxidized AQS on the delayed formation of biofilm, AQS pre-reduction was conducted before BES setup and the results show that the delay in the mid-log phase and the decrease in biofilm biomass at high AQS concentration disappeared (Figure 2D and Supplementary Figure 4), which further support the aforementioned hypothesis.

### Synergistic mechanism of AQS in promoting biofilm formation and EET rate

9,10-Anthraquinone-2-sulfonic acid is traditionally considered an ES, and the effect of ES on the EET rate has been explored in previous studies. However, the dual ability of AQS to promote both the EET rate of each cell and the biofilm formation rate was identified in this study.

Exogenous AQS enhanced the electron transfer rate from cells to the electrode compared with that in the system without AQS. As shown in Supplementary Figure 7, the biocurrent normalized by the biofilm biomass, that is, the biocurrent generated per microgram of total biofilm protein, increased with the increase in AQS concentration. Meanwhile, after 1,000 μM, the normalized current no longer increases, which indicates that there is a maximum limit value in the ability of AQS to mediate electron transfer. Moreover, the results in Supplementary Figure 8 show that the biocurrent density dropped sharply from 1.6 to 0.06 A m^−2^ when the original medium was replaced with a fresh medium without AQS. Although the biofilm mass decreased slightly after medium replacement (Supplementary Figure 9), the decreasing amplitude was much less than the reduction in current. These results revealed that AQS contributed markedly to biocurrent generation.

However, in addition to its role in EET acceleration, exogenous AQS also enhanced the biofilm biomass. The enhancement of biofilm formation and electron transfer synergistically affected biocurrent generation, which enhanced power generation. The addition of exogenous AQS substantially increased the biofilm total protein (Figure 2B), and the biocurrent density had a positive and linear correlation with the biofilm total protein (*R*^2^ = 0.97, standardized residual = 0.25) (Figure 5C). This strongly suggested that the increase in biomass markedly contributed to the increase in biocurrent generation. Meanwhile, due to the increase in AQS concentration, the electron transfer rate of each cell in high-concentration treatments (≥500 μM) increased (Supplementary Figure 7). The increasing EET rate probably increased ATP generation, thus enhancing the biofilm growth rate. Therefore, the slope of the current rise in high-concentration BESs increased. For the cells in suspension, the distance between the electrode and the cells in suspension (>100 μm) was longer than that between the electrode and the cells in the biofilm (< 10 μm), which considerably decreased the diffusion efficiency. Therefore, cells in biofilm instead of cells in suspension dominated the biocurrent production.

Hence, the enhancement of the biocurrent can be attributed to the synergistic mechanism associated with the biofilm formation and electron transfer rate, which are all related to the concentration of AQS.

### Physicochemical constraints on the *I*_max_ of AQS-mediated EET

Exogenous AQS significantly increased biocurrent density, and hence, the addition of ESs can be used as an effective strategy to increase biocurrent generation. However, as shown in the data in this study, *I*_max_ did not always increase with a rise in AQS concentration, possibly because the biocurrent output was limited by some specific physicochemical factors. The key factors in the system of EET are described in Supplementary Figure 10; they include biofilm biomass (Supplementary Figure 10A), abiotic process (Supplementary Figure 10B), and biotic process (Supplementary Figure 10C), and each plays a critical role under different conditions.

First, the biofilm biomass on the electrode (Supplementary Figure 10A) is possibly a critical factor that limited the biocurrent density in the system without ES. This is because, as shown in the system without AQS (Figures 1A, 2A), very few cells were attached to the electrode, which led to the generation of extremely low biocurrent. In addition, the biocurrent density showed a positive and linear correlation with the biofilm total protein on day 4 (*R*^2^ = 0.84) (Figure 5C), suggesting that the biomass is a limitation of the biocurrent output. However, with the biofilm covering the entire electrode, the biofilm biomass in different treatments becomes similar, and biofilm biomass may no longer be a key influencing factor.

Second, after the formation of a mature biofilm, the abiotic process involving the transport of electrons to the electrode is essential and should be considered (Supplementary Figure 10B). The diffusion rate of AQS has the potential to be a factor limiting *I*_max_. The biocurrent generated by diffusion can be calculated using Equation (1).


(1) $$\begin{array}{l} j=nF\left(\frac{D_{\text{AQ}S}\Delta \left[\text{AQ}{\text{S}}_{\text{red}}\right]}{\Delta z}\right) \end{array}$$

where *j* is the biocurrent density (A m^−2^), *D*_AQS_ is the diffusion coefficient of the AQS (m^2^·s^−1^), Δ*z* is the diffusion distance (m), Δ[AQS_red_] is the concentration gradient of AQS_red_ (mol·m^−3^), and *nF* is a conversion factor from moles to coulombs. According to a previous study, using *D*_AQS_ = 6.7 × 10^−10^·m^2^·s^−1^, Δ[AQS_red_] = 1 μM, and *n* = 2 for calculation (Torres et al., 2010), the theoretical maximum biocurrent density calculated with Equation (1) was 0.13 A·m^2^, which was lower than *I*_max_ (5.4 A·m^2^) in this study. However, increasing the AQS concentration can increase the concentration gradient (Δ[AQS_red_]), thus enhancing the EET rate of each cell. Using Δ[AQS_red_] = 50 μM, the calculated *I*_max_ was 6.5 A·m^2^, which was higher than *I*_max_ (5.4 A·m^2^). Meanwhile, no linear correlation was observed between *I*_max_ in Figures 1, 4 and the AQS concentration (data not show), suggesting that diffusion is not a rate-limiting step in BESs with a high concentration of AQS.

Finally, the most likely factor to limit *I*_max_ in AQS-mediated EET after the formation of a mature biofilm is the microbial AQS reduction rate (Supplementary Figure 10C). 9,10-Anthraquinone-2-sulfonic acid was reduced and lactate was oxidized in the biofilm during the EET of MR-1, which can be represented by the following reactions:


$$\begin{array}{l} {\text{C}}_{\text{3}}{\text{H}}_{\text{5}}{{\text{O}}_{\text{3}}}^{-}+\text{2}{\text{H}}_{\text{2}}\text{O}\overset{\text{MR-1}}{\underset{}{\to }}{\text{C}}_{\text{2}}{\text{H}}_{\text{3}}{{\text{O}}_{\text{2}}}^{-}+\text{HC}{{\text{O}}_{\text{3}}}^{-}+\text{5}{\text{H}}^{+}+\text{4}{\text{e}}^{-} \end{array}$$

                      Rxn (1)


$$\begin{array}{l} \text{AQS}+2{\text{H}}^{+}+2{\text{e}}^{-}\overset{\text{MR}-1}{\underset{}{\to }}\text{A}{\text{H}}_{2}QS \end{array}$$

                    Rxn (2)

The total reaction is as follows:


$$\begin{array}{l} \text{2}{\text{C}}_{\text{3}}{\text{H}}_{\text{5}}{{\text{O}}_{\text{3}}}^{-}+\text{4}{\text{H}}_{\text{2}}\text{O}+5\text{AQS}+\text{2}{\text{e}}^{-}\;\overset{\text{MR-1}}{\underset{}{\to }}\text{2}{\text{C}}_{\text{2}}{\text{H}}_{\text{3}}{{\text{O}}_{\text{2}}}^{-}\\ +2\text{HC}{{\text{O}}_{\text{3}}}^{-}+\text{5A}{\text{H}}_{\text{2}}\text{QS Rxn }\left(3\right) \end{array}$$

As shown in Rxn 3, the reduction in AQS can be considered a microbial catalytic process. Herein, lactate and AQS were the substrates and *S. oneidensis* MR-1 can be considered an enzyme. The concentration of lactate was high (30 mM), and hence, the Michaelis–Menten equation can be used to describe the kinetic energy of AQS microbial catalytic reaction. If we regard the biocurrent as the catalytic rate, once the biocurrent and the AQS concentration can be fitted by the Michaelis–Menten equation, it could indicate that the production of the biocurrent is determined by the reduction reaction of AQS. The Michaelis–Menten equation is given as follows:


(2) $$\begin{array}{l} v=\frac{V_{\max}\left[S\right]}{K_{m}+\left[S\right]} \end{array}$$

where [*S*] is the concentration of the rate-limiting substrate (corresponding to the concentration of AQS), *V*_max_ is the maximum catalytic rate (corresponding to *I*_max_), and *K*_m_ is the half-saturation constant.

**Figure 6:**
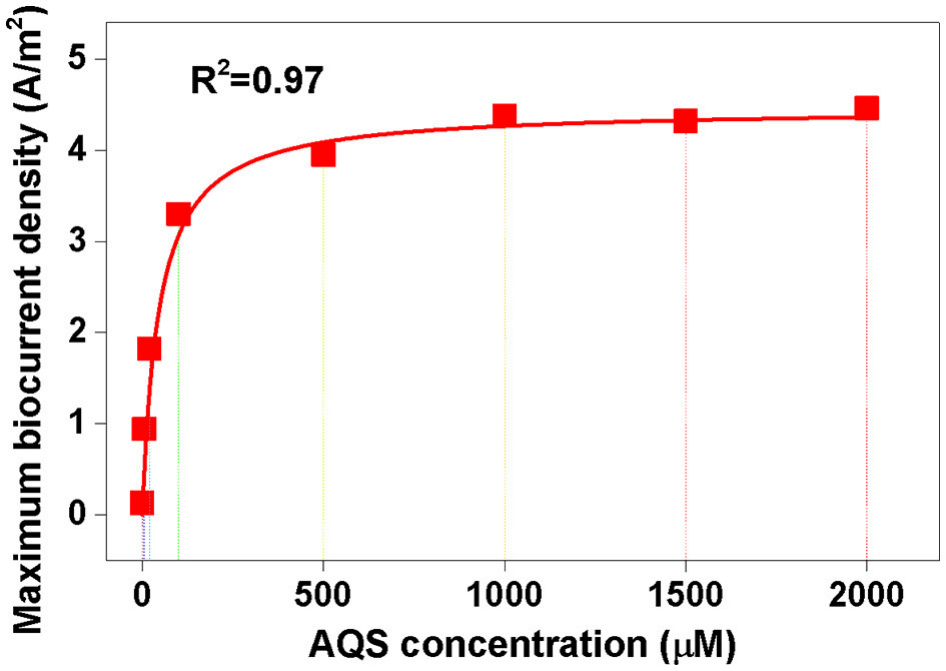
Michaelis–Menten equation fitting. The maximum biocurrent density was derived from the data in Figure 4B.

For Michaelis–Menten fitting, *I*_max_-values under different AQS concentrations in Figure 4B are shown in Figure 6. In this case, a pre-incubated biofilm ensures that the biomass was consistent for all the treatments. A high correlation with an *R*^2^-value of 0.97 and a low standardized residual of 0.31 was observed (Figure 6), which indicated that the biocurrent generation reaction in the BES was a typical enzymatic reaction. Hence, when the substrate concentration (AQS concentration) reached saturation, the catalytic rate no longer increased. 9,10-Anthraquinone-2-sulfonic acid reduction kinetics also showed that there was no significant increase in AQS bio-reduction rate when the AQS concentration increased from 500 to 1,000 μM (Supplementary Figure 3A), which was consistent with the results for the biocurrent density in Figure 6. These results indicated that the biocurrent generation reaction in the BES conforms to the laws of typical enzyme-catalyzed reactions. In other words, the biocatalytic reaction of AQS is the rate-limiting step.

The results of this study showed that the microbial catalysis efficiency, and not the diffusion rate, is the key limiting factor of *I*_max_ in a BES with mature biofilms. Despite a previous suggestion that the diffusion process of ESs is the rate-determining step in an electrochemical system of low ES concentrations (< 1 μM) (Torres et al., 2010), the results of this study demonstrated that the rate of AQS bio-reduction determined the maximum biocurrent density in a BES with high ES concentrations. Therefore, enhancing the metabolism rate of MR-1 using synthetic biology to increase the ES reduction rate or by discovering new ESs that are easily reduced by cells are potential strategies that can be used to further increase biocurrent generation.

### Environmental implications

The genus *Shewanella* is widely distributed in natural environments and has been used in artificial bio-energy and sewage treatment systems (Hau and Gralnick, 2007; Fredrickson et al., 2008). The importance of exogenous shuttles to *Shewanella* has been controversial. This study demonstrated that the growth of *S. oneidensis* MR-1 on an electrode is highly dependent on electron shuttling. Although the view that “direct EET of *S. oneidensis* MR-1 is highly efficient” was supported by the *in vitro* evidence of rapid electron exchange in an artificial cytochrome complex (MtrCAB) (White et al., 2013), whole-cell research of *S. oneidensis* MR-1 has shown extremely low biocurrent production without self-secretion of flavin (Kotloski and Gralnick, 2013). This supports the view that the shuttle is very critical. In this study, we demonstrated that biocurrent generation and biofilm growth were enhanced with the addition of exogenous ESs compared with that in a system without ES, which further confirmed the necessity of ESs in the EET of *Shewanella*.

## Conclusion

**Figure 7:**
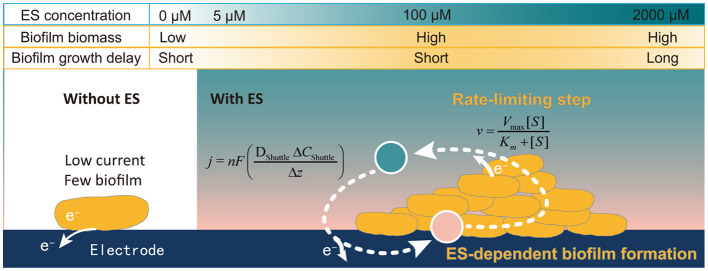
Schematic representation of the ES concentration-dependent biofilm formation process.

In this study, the quantification relationship between AQS concentration, biocurrent generation, and biofilm formation in *S. oneidensis* MR-1 was systematically analyzed. A new mechanism was identified, as described in Figure 7. First, biofilm formation by *S. oneidensis* MR-1 on an electrode was highly dependent on electron shuttling. Exogenous AQS dramatically enhanced the total biofilm biomass, whereas high concentrations of AQS (≥1,000 μM) delayed biofilm formation, which could be attributed to the beneficial effects of AQS on planktonic cell lysis. Second, the AQS bio-reduction rate is the most important factor limiting the *I*_max_ of AQS-mediated BESs. The maximum biocurrent density in the BES with different concentrations of AQS was strictly fitted to the Michaelis–Menten equation (*R*^2^ = 0.97). By revealing the mechanism, this study provided a simple strategy for enhancing electroactive biofilm formation in BESs using exogenous ES, especially for the prompt start-up of MFCs. This has important guiding significance for the enrichment of electroactive biofilms for wastewater treatment. Furthermore, it inspired us to question whether ESs have a similar role in regulating the growth of electroactive biofilms in a natural system. The contribution of ESs toward the maintenance of the biofilm microbial community in sludge and sediments requires further clarification in future studies.

## Data availability statement

The original contributions presented in the study are included in the article/Supplementary material, further inquiries can be directed to the corresponding author/s.

## Author contributions

YW, FL, and TL conceived and designed the experiments. XZ, FD, WL, and YW were responsible for drafting the article. XZ, FD, ML, XW, and YW were involved in the experiments and data analysis. All authors contributed to the article and approved the submitted version.
