# Supplementary material for: Electron shuttle-dependent biofilm formation and biocurrent generation: Concentration effects and mechanistic insights
Source: Front Microbiol. 2023 Mar 1;14:1070800. doi: 10.3389/fmicb.2023.1070800 (PMC10016380; doi:10.3389/fmicb.2023.1070800)
Supplement: Supplementary file 1 [file Data_Sheet_1.doc]

**Supporting Information**

**Electron shuttle-dependent biofilm formation and biocurrent generation: concentration effect and mechanistic insight**

Xiao Zhu^1,2^,^3,4†^, Fei Dou^2,3†^, Mingliang Long^2,3,5^, Xinxin Wang^2,3,5^, Wei Liu^5^, Fangbai Li^2,3^, Tongxu Liu^2,3^, Yundang Wu^2,3*^

^1^ *Guangzhou Institute of Geochemistry, Chinese Academy of Sciences, Guangzhou 510640, China*

^2^ *National-Regional Joint Engineering Research Center for Soil Pollution Control and Remediation in South China, Guangzhou, China*

^3^ *Guangdong Key Laboratory of Integrated Agro-environmental Pollution Control and Management, Institute of Eco-environmental and Soil Sciences, Guangdong Academy of Sciences, Guangzhou, China*

^4^ *University of Chinese Academy of Sciences, Beijing, China*

^5^ *College of Materials and Energy, South China Agricultural University, Guangzhou, China*

* *Corresponding author.*

*Tel.:+86 20 87025180; Fax: +86 20 87024123.*

*Email: ydwu@soil.gd.cn*

***Frontiers in Microbiology***

**Table S1** Compositions of the vitamin and mineral bulk solution

| **Vitamins** | **Concentration (mg·L^-1^)** |
| --- | --- |
| Biotin | 2.0 |
| Folic acid | 2.0 |
| Pyridoxine hydrochloride | 10.0 |
| Riboflavin | 5.0 |
| Thiamine | 5.0 |
| Nicotinic acid | 5.0 |
| Pantothenic acid | 5.0 |
| B-12 | 0.1 |
| *p*-Aminobenzoic acid | 5.0 |
| Thioctic acid | 5.0 |

| **Minerals** | **Concentration (g·L^-1^)** |
| --- | --- |
| Na_2_WO_4_·2H_2_O | 0.025 |
| Na_2_MoO_4_ | 0.025 |
| NiCl_2_·6H_2_O | 0.024 |
| H_3_BO_3_ | 0.01 |
| CuSO_4_·5H_2_O | 0.01 |
| AlK(SO_4_)_2_·12H_2_O | 0.01 |
| CaCl_2_ | 0.1 |
| CoCl_2_·6H_2_O | 0.1 |
| FeSO_4_·7H_2_O | 0.1 |
| ZnSO_4_·7H_2_O | 0.1 |
| MnSO_4_·H_2_O | 0.5 |
| NaCl | 1.0 |
| MgSO_4_·7H_2_O | 3.0 |
| Trisodium nitrilotriacetic acid | 1.5 |

**
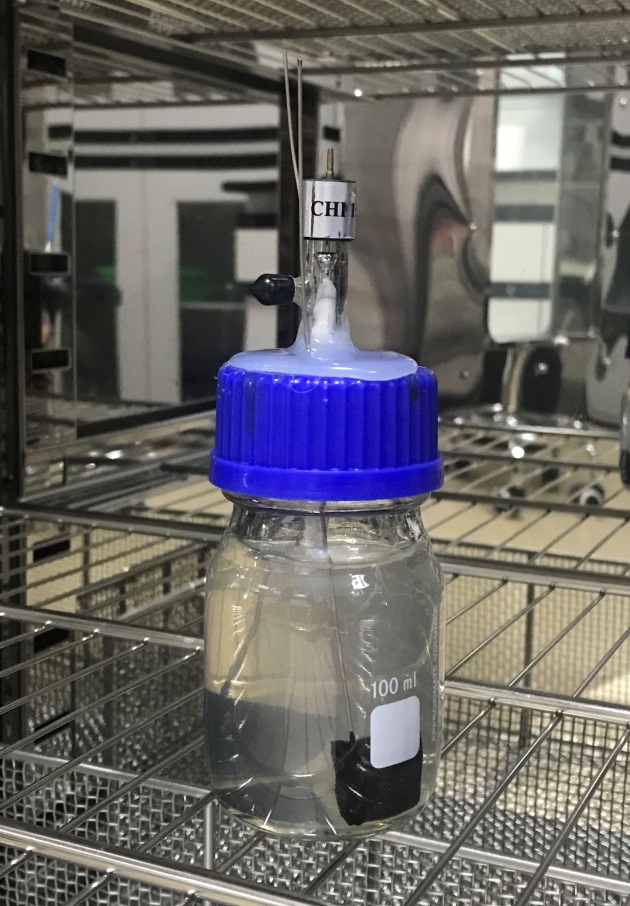

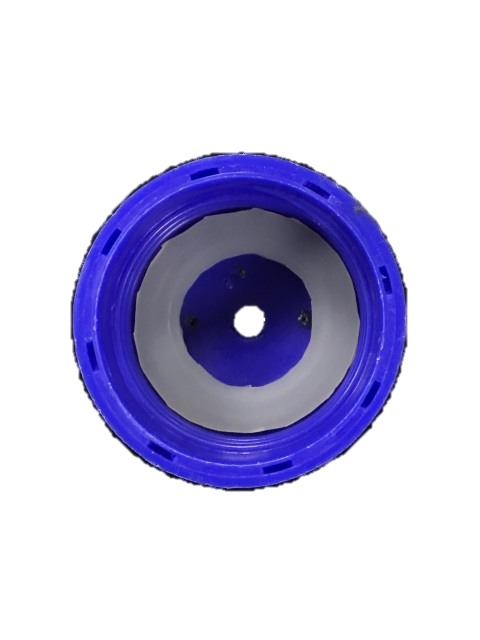
**

**Figure S1** Photo of a bioelectrochemistry system (BES). Carbon cloth was used as working and counter electrodes and a calomel electrode was used as a reference. The cap was sealed with a silicone ring mat and hot melt adhesive.


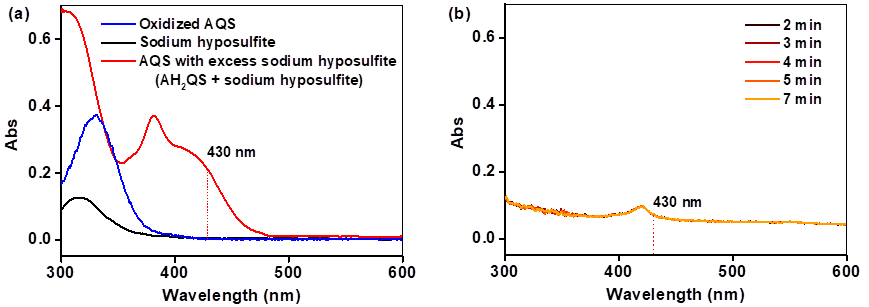


**Figure S2** (a) Spectra of oxidized 9,10-anthraquinone-2-sulfonic acid (AQS), reduced AQS (AH_2_QS), and the reducer sodium hyposulfite. (b) Spectra of the bacteria. It was stable at 430 nm; thus, the absorbance of bacteria could be subtracted as a baseline.


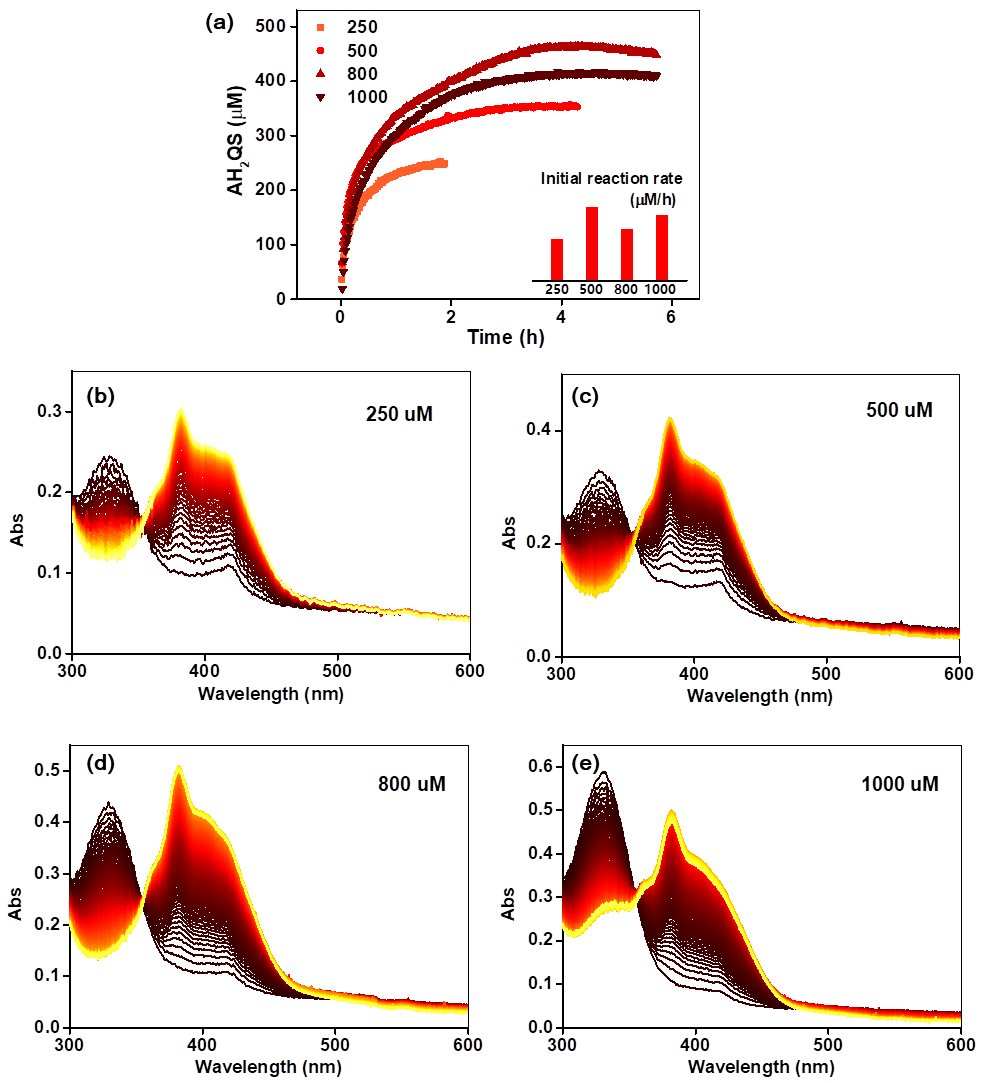


**Figure S3** (a) The AH_2_QS producing kinetics at different AQS concentration. The inset shows the initial reaction rate calculated by the rate constant of pseudo-first-order kinetics and the initial AQS concentration. (b–e) UV/Vis diffuse-transmittance absorption spectra of the AQS reduction process. The four figures represent the treatments with different AQS concentrations.

**Figure S4** The first derivative analysis of the current results in Figure 4(a). The AQS was pre-reduced before BESs setup.

**Figure S5** The biofilm biomass on electrodes. The potentiostatic incubation in BESs was started after the AQS pre-reduction. The corresponded current density is shown in Figure 4a. For better comparison, all the biomass values were normalized to the biofilm total protein of the biofilm in 2000 μM AQS.

**Figure S6** The biofilm biomass on electrodes. The biofilms were all incubated in BESs with 50 μM of AQS for 4 days. Then, the original medium in BESs was replaced by a new medium with different concentration of pre-reduced AQS. The corresponded current density is shown in Figure 4b. For better comparison, all the biomass values were normalized to the biofilm total protein of the biofilm in 2000 μM AQS.

**Figure S7** The maximum current shown in Figure 1 normalized by the biofilm biomass.


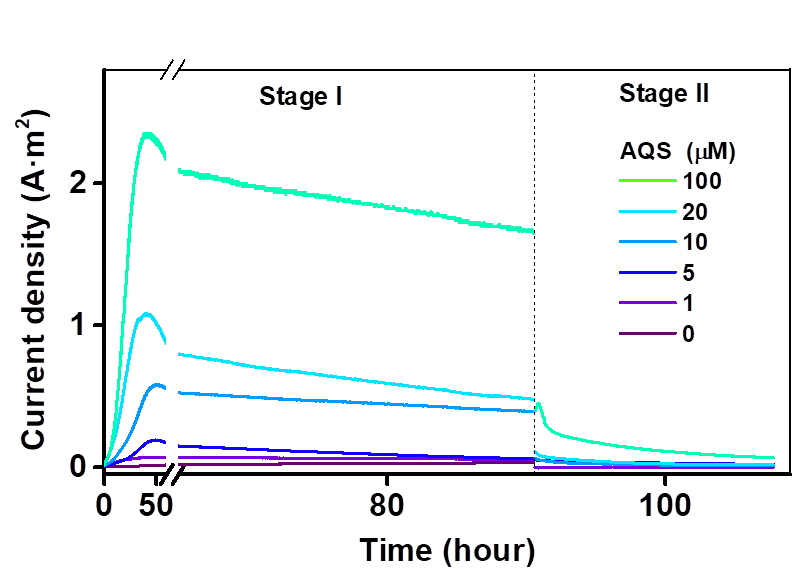


**Figure S8** Current density (*I*, A m^−2^) in BESs over time with/without AQS during two different stages of operation. Stage I, the medium with different concentration of AQS and *S. oneidensis* MR-1 cells was added into the BESs and then incubated potentiostatically for 4 days; stage II, the original medium were replaced by a new medium without AQS.

**Figure S9** Biofilm total protein before and after the medium replacement shown in Figure S8. For better comparison, all the biomass values were normalized to the biofilm total protein of the biofilm in 100 μM AQS before medium replacement.


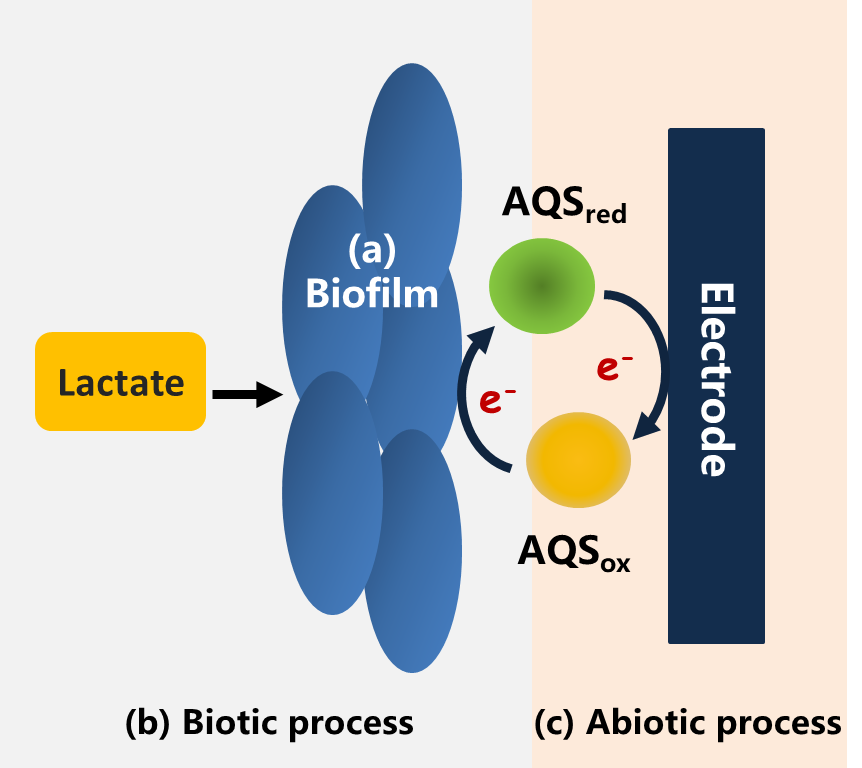


**Figure S10** The schematic of the biocurrent generation process. (a) Biofilm; (b) The biotic AQS reduction process; (c) The abiotic reduction of AQS on the electrode. Figure
